# No temporal contrast enhancement of simple decreases in noxious heat

**DOI:** 10.1152/jn.00335.2018

**Published:** 2019-03-06

**Authors:** Brianna Beck, Sahana Gnanasampanthan, Gian Domenico Iannetti, Patrick Haggard

**Affiliations:** ^1^Institute of Cognitive Neuroscience, University College London, London, United Kingdom; ^2^Medical School, University College London, London, United Kingdom; ^3^Department of Neuroscience, Physiology and Pharmacology, University College London, London, United Kingdom

**Keywords:** nociception, offset analgesia, pain, psychophysics, signal detection

## Abstract

Offset analgesia (OA) studies have found that small decreases in the intensity of a tonic noxious heat stimulus yield a disproportionately large amount of pain relief. In the classic OA paradigm, the decrease in stimulus intensity is preceded by an increase of equal size from an initial noxious level. Although the majority of researchers believe this temporal sequence of two changes is important for eliciting OA, it has also been suggested that the temporal contrast mechanism underlying OA may enhance detection of simple, isolated decreases in noxious heat. To test whether decreases in noxious heat intensity, by themselves, are perceived better than increases of comparable sizes, we used an adaptive two-interval alternative forced choice task to find perceptual thresholds for increases and decreases in radiant and contact heat. Decreases in noxious heat were more difficult to perceive than increases of comparable sizes from the same initial temperature of 45°C. In contrast, decreases and increases were perceived equally well within a common range of noxious temperatures (i.e., when increases started from 45°C and decreases started from 47°C). In another task, participants rated the pain intensity of heat stimuli that randomly and unpredictably increased, decreased, or remained constant. Ratings of unpredictable stimulus decreases also showed no evidence of perceptual enhancement. Our results demonstrate that there is no temporal contrast enhancement of simple, isolated decreases in noxious heat intensity. Combined with previous OA findings, they suggest that long-lasting noxious stimuli that follow an increase-decrease pattern may be important for eliciting the OA effect.

**NEW & NOTEWORTHY** Previous research suggested that a small decrease in noxious heat intensity feels surprisingly large because of sensory enhancement of noxious stimulus offsets (a simplified form of “offset analgesia”). Using a two-alternative forced choice task where participants detected simple increases or decreases in noxious heat, we showed that decreases in noxious heat, by themselves, are no better perceived than increases of comparable sizes. This suggests that a decrease alone is not sufficient to elicit offset analgesia.

## INTRODUCTION

Offset analgesia (OA) is a phenomenon whereby a small decrease in the intensity of tonic noxious heat stimulation causes a disproportionately large reduction in perceived pain level. The first study to systematically investigate OA showed that a 1°C drop in the intensity of a contact heat stimulus yielded the same amount of pain relief as a 15°C drop (Grill and Coghill 2002). Since then, several studies have investigated the possible mechanisms of OA (Derbyshire and Osborn 2008, 2009; Honigman et al. 2013; Martucci et al. 2012a, 2012b; Mørch et al. 2015; Nahman-Averbuch et al. 2014; Naugle et al. 2013; Niesters et al. 2011a, 2011b; Nilsson et al. 2014; Oudejans et al. 2015; Petre et al. 2017; Yelle et al. 2008, 2009). Some of those studies explained OA as the product of a temporal filtering mechanism that enhances detection of noxious stimulus offsets (Grill and Coghill 2002; Mørch et al. 2015; Yelle et al. 2008, 2009).

Importantly, most studies investigating OA used tonic noxious heat stimuli with a particular stimulation profile: the stimulus started at an initial level of noxious heat, was increased to an even higher level, and then decreased either back to the initial noxious level or to a temperature well below the initial one and outside the noxious range (Derbyshire and Osborn 2008, 2009; Grill and Coghill 2002; Honigman et al. 2013; Martucci et al. 2012a, 2012b; Nahman-Averbuch et al. 2014; Naugle et al. 2013; Niesters et al. 2011a, 2011b; Nilsson et al. 2014; Oudejans et al. 2015; Petre et al. 2017; Yelle et al. 2008, 2009). Whereas most researchers within the field believe that this dynamic increase-decrease sequence is key to eliciting OA, a minority have suggested that temporal contrast enhancement might be a general process affecting perception of simple decrements in noxious heat stimulation, rather than just long-lasting stimuli following a particular dynamic sequence. Indeed, a few studies found evidence for enhanced perception of simple, isolated decreases in noxious heat intensity that were not preceded by increases from an initial noxious heat level or by prolonged noxious stimulation at a constant temperature (Mørch et al. 2015; Yelle et al. 2008). It thus remained unclear whether an increase in temperature from an initial noxious level was important for eliciting temporal contrast enhancement of the subsequent decrease. Moreover, all these previous studies used pain ratings to measure perceived changes in noxious heat intensity. Such ratings could potentially be influenced by nonsensory processes, such as biases in using the rating scale, and would therefore be unsatisfactory for testing whether there is enhanced sensory processing of simple decreases in noxious heat.

To provide a more rigorous test of whether decreases in noxious heat intensity, by themselves, are perceptually enhanced relative to increases of comparable sizes, we used a two-interval alternative forced choice task (2IFC) coupled with a staircase procedure to find the smallest detectable increase and decrease in noxious heat (i.e., increase and decrease detection thresholds; *experiments 1–3*). We used a similar procedure to find the smallest discriminable difference between two increases or decreases of different magnitudes (i.e., increase and decrease discrimination thresholds; *experiment 1*). Such a procedure assesses perception of changes in noxious heat intensity while minimizing bias. However, to better compare our results with previous findings, we also presented single noxious heat stimuli that either decreased from 47°C to 46°C, increased from 46°C to 47°C, or remained constant at either 46°C or 47°C. Participants rated the intensity of the pain they felt at the end of each stimulus, after it had reached its final temperature (*experiment 2*). Based on a previous study that found temporal contrast enhancement of decreases in noxious heat compared with increases when the two were presented separately (Mørch et al. 2015), we expected to find smaller detection thresholds for decreases than for increases, because sharper temporal filtering of decreases should make them easier to detect. Conversely, we predicted larger discrimination thresholds for decreases of different sizes than for increases of different sizes, because of previous evidence that even a 1°C drop in noxious heat feels as large as a 15°C drop (Grill and Coghill 2002). Additionally, we expected lower pain intensity ratings of a 46°C temperature that followed a drop from 47°C, compared with a stimulus that stayed at a constant 46°C temperature.

## METHODS

### Participants

Sixteen healthy volunteers were recruited for each experiment through the participant database of the Institute of Cognitive Neuroscience at University College London (UCL). The sample size was determined using G*Power 3.1.9.2 (Faul et al. 2007) and was based on the number of participants needed per experiment to achieve a power of 0.80 with an estimated temporal contrast enhancement effect size (Cohen’s *d_z_*) of 0.76 (Grill and Coghill 2002). Four men and 12 women (mean age = 23 yr; range = 19–29 yr) participated in *experiment 1*. A separate group of 6 men and 10 women (mean age = 25 yr; range = 18–34 yr) participated in *experiment 2*. Another separate group of 8 men and 8 women (mean age = 28 yr, range = 20–38 yr) participated in *experiment 3*. Eligibility criteria included being 18–40 yr of age, not having sensitive skin or a dermatological condition, and not having taken any analgesic medications within 24 h before the experiment. All volunteers gave their written informed consent to participate in the experiments and were free to withdraw from the study at any point in time. Four participants in *experiment 2* and one in *experiment 3* opted to withdraw because they felt the stimuli were too painful. This possibility had been explicitly included in the protocol and was not considered an adverse event. Data from those participants were excluded from all statistical analyses, and we recruited additional participants to replace them. All procedures were approved by the UCL Research Ethics Committee and carried out in accordance with the Declaration of Helsinki. Participants were compensated for their time with a payment of £7.50 per hour.

### Apparatus and Materials

All experimental sessions were carried out in a testing room at the UCL Institute of Cognitive Neuroscience. A laptop computer running LabVIEW 2012 (National Instruments, Austin, TX) was used to run all tasks and record participant responses. Noxious stimuli consisted of either radiant or contact heat, and were delivered to the dorsum of the participant’s left hand.

Radiant heat stimuli were generated by a skin temperature feedback-controlled infrared CO_2_ laser (wavelength = 10.6 μm; SIFEC, Ferrières, Belgium), which allows selective activation of epidermal free nerve endings belonging to Aδ and C nociceptive afferents (Baumgärtner et al. 2005). The laser device continuously samples the skin temperature at the stimulation site so that it can adjust its output energy to reach and maintain the target temperature. Importantly, this device can deliver stimuli lasting several seconds and is thus optimal for exploring the perceptual correlates of relatively slow increases and decreases in nociceptive input (Mancini et al. 2016). The laser beam was transmitted through an optic fiber, and its diameter was set to 6 mm (28 mm^2^) by focusing lenses.

Contact heat stimuli were generated by a Peltier thermode (Physitemp, Clifton, NJ). The thermode probe had a round contact area (diameter = 13 mm). It was attached to a wood bar controlled by a high power servomotor that brought the probe into contact with the left hand dorsum at the beginning of each stimulus and then retracted it at the end of the stimulus. The probe was preheated to the starting temperature of the stimulus before being applied to the hand.

### Experiment 1

*Experiment 1* consisted of two sessions on separate days. Radiant heat stimuli were delivered in one session, and contact heat stimuli were delivered in the other session. Both sessions occurred at the same time of day to minimize the impact of diurnal variations in pain perception (Glynn and Lloyd 1976; Strian et al. 1989). Session order was counterbalanced across participants.

Each session comprised four tasks: decrease detection, decrease discrimination, increase detection, and increase discrimination. Each task, consisting of 30 trials, was carried out in a separate block. We determined the smallest change in temperature that could be detected (detection thresholds), as well as how precisely changes in temperature could be perceived (discrimination thresholds), using a 2IFC paradigm and an adaptive 3-down/1-up staircase procedure, which converges on a 79.4% accuracy threshold (Levitt 1971). Detection and discrimination thresholds were calculated by averaging the size of the increase or decrease in stimulus intensity across the last 20 trials of each block. The first 10 trials of each block, during which the staircase was still converging, were not included in the threshold determination.

Task order, with respect to increase and decrease thresholds, was counterbalanced across participants. The detection task was always done before the corresponding discrimination task so that the detection threshold could be used as the reference stimulus in the discrimination task. Breaks of ~5 min were given between blocks.

On every trial, two noxious heat stimuli were delivered to the left hand dorsum. Each stimulus lasted 6 s. At the beginning of the trial, participants pressed a key to initiate the first stimulus. At 3 s after the end of the first stimulus, participants pressed a key again to initiate the second stimulus. Key presses to initiate the stimuli were included as a safety precaution. The location of noxious heat stimulation was shifted by ~2 cm between stimuli to avoid peripheral effects such as receptor adaptation, vascular responses, and persistent changes in skin temperature. Throughout each trial, participants fixated a cross presented on the computer screen ~60 cm in front of them.

**Fig. 1.**
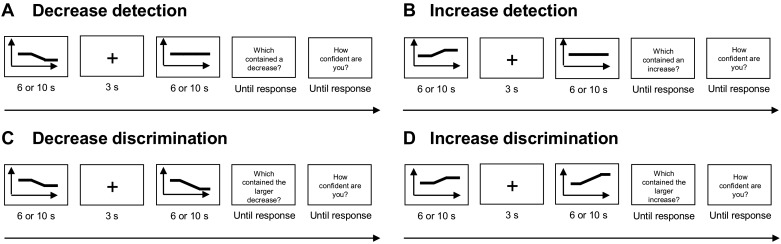
Example trials from each of the four 2-interval alternative forced choice (2IFC) tasks used in *experiment 1*. *A*: decrease detection blocks. *B*: increase detection blocks. *C*: decrease discrimination blocks. *D*: increase discrimination blocks. Decrease and increase detection blocks were also run in *experiments 2* and *3*. Noxious heat stimuli lasted 6 s each in *experiments 1* and *2*, and 10 s each in *experiment 3*.

Stimuli are illustrated in Fig. 1. In the decrease detection block, one stimulus remained at a constant temperature of 45°C for 6 s. The temperature of the other stimulus changed: it started at 45°C for 1 s, then decreased to 42.5°C at a rate of 2°C/s, and remained at 42.5°C for the rest of the 6-s stimulus duration. The temperature decrease was equally likely to appear in the first or the second stimulus of each trial. After the second stimulus, the computer screen displayed the question, “Which stimulus contained the decrease?” Participants pressed one key if they thought the decrease occurred in the first stimulus or another key if they thought it occurred in the second stimulus. Following a 3-down/1-up staircase procedure, the size of the temperature decrease on the following trial increased by 0.5°C (i.e., a larger temperature difference) after an incorrect answer and decreased by 0.5°C (i.e., a smaller temperature difference) after three successive correct answers. After answering the first question, participants were also asked “How confident are you about your answer?” They pressed one key for “confident” or another key for “just guessing.” The program then proceeded to the next trial (Fig. 1*A*).

The increase detection block followed the same procedure as the decrease detection block, except that the temperature of one stimulus in each trial increased at a rate of 2°C/s (from 45°C to 47.5°C on the first trial, and was then adjusted on subsequent trials following the same rules described above). As in the decrease detection block, the temperature of the other stimulus remained constant at 45°C for the entire 6-s duration. Participants had to report which of the two stimuli contained the temperature increase and then gave confidence judgments (Fig. 1*B*).

In the decrease discrimination block, participants had to detect which of the two stimuli contained a larger temperature decrease. Both stimuli started at an initial temperature of 45°C. The temperature of one stimulus decreased from 45°C to the participant’s previously determined decrease detection threshold, at a rate of 2°C/s. The temperature of the other stimulus decreased to 2.5°C below the participant’s decrease detection threshold. The larger decrease was equally likely to appear in the first or the second stimulus of each trial. After the second stimulus, the screen displayed the question, “Which stimulus contained the larger decrease?” Participants pressed one key if they thought the larger decrease occurred in the first stimulus or another key if they thought it occurred in the second stimulus. The size of the larger temperature decrease on the following trial increased by 0.5°C after an incorrect answer and decreased by 0.5°C after three successive correct answers. The larger decrease was always greater than the decrease detection threshold but never reached a temperature below 35°C. The size of the smaller temperature decrease was the same on every trial (i.e., it was equal to the decrease detection threshold). Participants also gave confidence judgments, as described above (Fig. 1*C*).

The increase discrimination block followed a similar procedure, except for the direction of stimulus temperature changes. The smaller temperature increase was always equal to the participant’s previously determined increase detection threshold. The larger temperature increase was initially 2.5°C higher than the increase detection threshold and was adjusted on subsequent trials following the same rules described above. The larger increase was always greater than the increase detection threshold, but it never increased beyond 50°C, for safety reasons. Participants reported which stimulus contained the larger temperature increase and gave confidence judgments (Fig. 1*D*). We could not estimate increase discrimination thresholds for five participants because it would have required increasing stimulus temperature above 50°C. Data from these five participants were excluded from the analysis of discrimination thresholds.

### Experiment 2

In *experiment 2*, we tested whether the findings of *experiment 1* (smaller detection thresholds for increases in noxious heat, compared with decreases) could be replicated. Procedures for finding perceptual thresholds were similar to those of *experiment 1*, with the following differences: *1*) we used only radiant heat stimuli, so the experiment was conducted in a single session; *2*) we measured detection thresholds, but not discrimination thresholds; *3*) the rate of temperature change was increased to 4°C/s so that we could test how well our findings generalize to different rates of temperature change; and *4*) stimuli that included an increase or decrease remained at the initial temperature of 45°C for 3 s (not for 1 s as in *experiment 1*) before any temperature change so that the initial and final plateau stages of the stimulus profile were more similar in duration. Block order (increase detection vs. decrease detection) was again counterbalanced across participants.

*Experiment 2* also included a separate task in which participants rated perceived pain intensity during noxious heat stimulation, using an electronic visual analog scale (eVAS). On each trial, a single radiant heat stimulus was presented. There were four different stimulus types: *1*) 46–46°C, where the stimulus temperature remained constant at 46°C for 6 s; *2*) 47–47°C, where the stimulus temperature remained constant at 47°C for 6 s; *3*) 46–47°C, where the stimulus temperature started at 46°C for 3 s, increased to 47°C at 4°C/s, and remained at 47°C for 2.75 s; and *4*) 47–46°C, where the stimulus temperature started at 47°C for 3 s, decreased to 46°C at 4°C/s and remained at 46°C for 2.75 s.

**Fig. 2.**
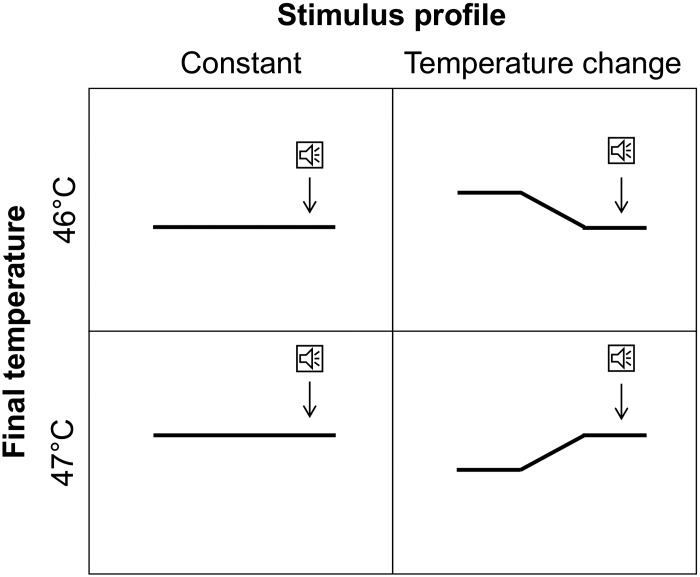
The 4 stimulus types delivered in the intensity rating task in *experiment 2*. Stimuli were delivered in a randomized order. Participants used an electronic visual analog scale (eVAS; 0–10) to rate the intensity of pain they felt at the time of an auditory tone presented 1 s before the end of the stimulus (arrows).

On each trial, participants pressed a key to initiate the stimulus. A transient auditory stimulus occurred 1 s before the end of the stimulus, after it had reached its final temperature. At the end of the stimulus, an eVAS appeared on the screen, ranging from 0 (no pain) to 10 (worst pain imaginable). Participants were asked to rate the intensity of the pain they felt at the time of the auditory stimulus (Fig. 2). Each type of stimulus was presented 14 times in a randomized order, for a total of 56 trials, divided into 2 blocks of 28 trials each. Participants were not given any instructions about the time courses of the stimuli (i.e., whether their temperature would increase, decrease, or stay the same). Task order (detection thresholds first or rating task first) was counterbalanced across participants. Breaks of ~5 min were given between blocks.

### Experiment 3

In *experiments 1* and *2*, detection thresholds were measured by increasing or decreasing temperature from a common initial level. Thus the staircases used for measuring increase detection necessarily involved higher temperatures than the staircases used for measuring decrease detection. This difference in the temperature ranges used could potentially explain the difference between increase and decrease detection thresholds, if there was a positively accelerating relation between stimulus temperature and perceived intensity. Indeed, previous studies have found such a stimulus-response function for noxious contact heat stimulation >42°C (Baliki et al. 2009; Coghill et al. 1993; Defrin and Urca 1996; Kenshalo et al. 1979; Nielsen et al. 2005; Price et al. 1978, 1983, 1994; Svensson et al. 1997).

Accordingly, *experiment 3* tested detection of decreases using noxious radiant heat stimuli that started from a higher initial temperature than that used to test detection of increases. This procedure aimed to find perceptual thresholds for increases and decreases in noxious heat by using overlapping temperature ranges for increase detection and decrease detection. Procedures for finding detection thresholds were similar to those of *Experiment 2*, with the following differences: *1*) decreasing stimuli started from a higher initial temperature of 47°C, whereas increasing stimuli still started from 45°C; *2*) stimulus duration was lengthened to 10 s; *3*) stimuli that included an increase or decrease remained at the initial temperature for 5 s before the temperature change; and *4*) confidence ratings were collected using a 4-point scale, with 1 as the minimum and 4 as the maximum, to allow participants to report finer differences in their confidence level. Block order (increase detection vs. decrease detection) was again counterbalanced across participants. As in *experiment 2*, the rate of temperature change was 4°C/s.

## RESULTS

### Experiment 1

#### Detection thresholds.

**Fig. 3.**
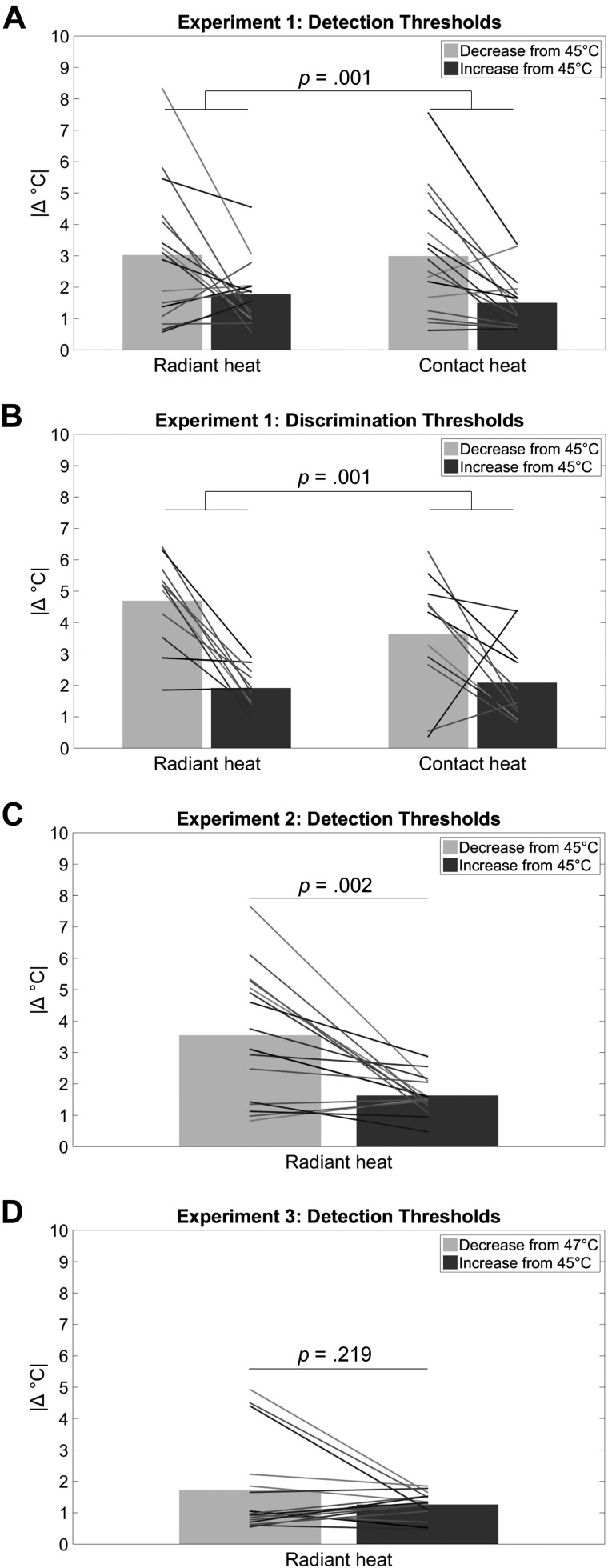
Perceptual thresholds for decreases and increases in radiant (laser) and contact (thermode) noxious heat intensity from *experiments 1* and *2*. Thresholds are represented as unsigned temperature magnitudes (|Δ°C|). Bars represent the mean thresholds, and lines represent single-participant thresholds. *A*: thresholds for detecting which of the 2 stimuli contained a decrease or increase from 45°C in *experiment 1*. *B*: thresholds for discriminating which stimulus contained the larger decrease or increase from 45°C in *experiment 1*. *C*: thresholds for detecting which of the 2 stimuli contained a decrease or increase from 45°C in *experiment 2*. *D*: thresholds for detecting which of the 2 stimuli contained a decrease from 47°C or an increase from 45°C in *experiment 3*.

A 2 × 2 repeated-measures ANOVA with the factors direction (two levels: increase or decrease in stimulus intensity) and stimulus type (two levels: radiant or contact heat) was run on detection thresholds. There was a main effect of direction [*F*(1,15) = 16.43, *P* = 0.001, $\eta_{\text{p}}^{2}$ =0.52], with larger detection thresholds for decreases in noxious heat intensity [mean (M) = 3.01°C, SE =  ±0.43°C] than for increases (M = 1.64°C, SE =  ±0.21°C). There was no main effect of stimulus type [*F*(1,15) = 0.38, *P* = 0.549, $\eta_{\text{p}}^{2}$ = 0.02] and no interaction [*F*(1,15) = 0.14, *P* = 0.709, $\eta_{\text{p}}^{2}$ = 0.01; Fig. 3*A*].

#### Discrimination thresholds.

Another 2 × 2 repeated-measures ANOVA with the factors direction (increase or decrease in stimulus intensity) and stimulus type (radiant or contact heat) was run on discrimination thresholds. Again, there was a main effect of direction [*F*(1,10) = 19.02, *P* = 0.001, $\eta_{\text{p}}^{2}$ = 0.66], with larger thresholds for discriminating between two decreases in noxious heat intensity (M = 4.16°C, SE =  ±0.39°C) than for discriminating between two increases (M = 2.00°C, SE =  ±0.24°C). There was no main effect of stimulus type [*F*(1,10) = 1.14, *P* = 0.310, $\eta_{\text{p}}^{2}$ = 0.10] and no interaction [*F*(1,10) = 3.08, *P* = 0.110, $\eta_{\text{p}}^{2}$ = 0.24; Fig. 3*B*].

#### Confidence judgments.

We were interested in whether confidence judgments would differ between increases and decreases in noxious heat intensity, after accounting for any effects of accuracy and task difficulty on confidence. To this end, we ran a mixed logit model (Jaeger 2008) for binomially distributed outcomes (1 = confident, 0 = just guessing) with random intercepts by participant, using the generalized linear mixed effects model function in R package “lme4” (Bates et al. 2015). The categorical fixed effects were task (1 = detection, 0 = discrimination), stimulus type (1 = radiant heat, 0 = contact heat), direction (1 = increase, 0 = decrease), and trial-by-trial accuracy (1 = correct, 0 = incorrect). There was one continuous fixed effect: the size of the temperature change, or, in discrimination blocks, the size of the difference between the two temperature changes (rescaled so that 1 = maximum change/difference across participants, 0 = no change/difference). We report the marginal significance of each fixed effect with the other fixed effects in the model.

Unsurprisingly, accuracy predicted higher confidence judgments, β = 1.12, SE =  ±0.09, *P* = 2 × 10^−16^, as did the size of the temperature change (or the difference between the two temperature changes; β = 1.01, SE =  ±0.29, *P* = 0.0004). Task was also a significant predictor of confidence judgments, with higher confidence in detection judgments than discrimination judgments (β = 0.19, SE =  ±0.08, *P* = 0.020). Finally, increases predicted higher confidence judgments than decreases, even after trial-by-trial variability in accuracy and difficulty were accounted for (β = 0.67, SE =  ±0.09, *P* = 5 × 10^−13^). Stimulus type (radiant or contact heat) was not a significant predictor of confidence (β = 0.07, SE =  ±0.08, *P* = 0.374).

### Experiment 2

#### Detection thresholds.

A paired-samples *t*-test showed that detection thresholds were larger for decreases in noxious heat intensity (M = 3.55°C, SE =  ±0.52°C) than for increases (M = 1.63°C, SE =  ±0.15°C) [*t*(15) = 3.82, *P* = 0.002, Cohen’s *d_z_* = 0.95], replicating the result from *experiment 1* (Fig. 3*C*).

#### Confidence judgments.

We used a mixed logit model with random intercepts by participant to analyze confidence judgments, as in *experiment 1* (see results, *Experiment 1*, *Confidence judgments*). Again, accuracy predicted higher confidence judgments (β = 1.04, SE =  ±0.17, *P* = 1 × 10^−9^). However, neither the size of the temperature change (β = 0.87, SE =   0.61, *P* = 0.157) nor its direction (β = 0.25, SE =   0.17, *P* = 0.156) was a significant predictor of confidence.

#### Pain intensity ratings.

**Fig. 4.**
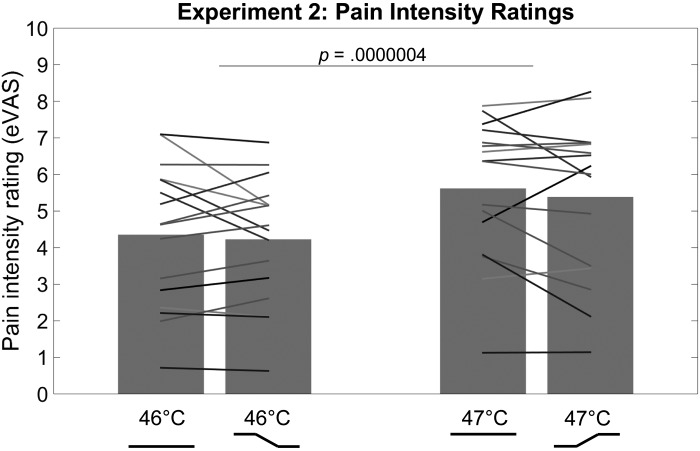
Pain intensity ratings on a 0–10 electronic visual analog scale (eVAS). Participants were instructed to rate the intensity of pain they felt at the time of an auditory tone presented 1 s before the end of the stimulus, when all stimuli had reached their final temperatures. Bars represent the mean ratings across participants, and lines represent the mean ratings of each participant.

A 2 × 2 repeated-measures ANOVA with the factors stimulus profile (constant or variable) and final stimulus temperature (46°C or 47°C) was run on pain intensity ratings. There was a main effect of final stimulus temperature [*F*(1,15) = 72.66, *P* = 0.0000004, $\eta_{\text{p}}^{2}$ = 0.83], with higher pain intensity ratings of stimuli ending at 47°C (M = 5.50, SE =  ±0.49) than stimuli ending at 46°C (M = 4.29, SE =  ±0.45). There was no main effect of stimulus profile [*F*(1,15) = 1.49, *P* = 0.241, $\eta_{\text{p}}^{2}$ = 0.09] and no interaction [*F*(1,15) = 0.12, *P* = 0.737, $\eta_{\text{p}}^{2}$ = 0.01; Fig. 4].

### Experiment 3

#### Detection thresholds.

The mean threshold for detecting a decrease in noxious heat was 1.72°C (i.e., a drop from 47°C to 45.28°C; SE =  ±0.38°C), and the mean threshold for detecting an increase was 1.26°C (i.e., a rise from 45°C to 46.26°C; SE =  ±0.10°C). These threshold values indicate that our design successfully produced overlapping temperature ranges for testing increase and decrease detection thresholds. In contrast to *experiments 1* and *2* (Fig. 3, *A* and *C*), a paired-samples *t*-test showed no effect of temperature change direction on detection thresholds in *experiment 3* [*t*(15) = 1.28, *P* = 0.219, Cohen’s *d_z_* = 0.32; Fig. 3*D*].

#### Confidence ratings.

To analyze confidence ratings on a four-point scale, we ran a mixed ordered logit model with random intercepts by participant, using the cumulative link mixed model function in R package “ordinal” (Christensen 2015). As in *experiments 1* and *2*, accuracy predicted higher confidence judgments (β = 1.55, SE =  ±0.16, *P* = 2 × 10^−16^). Larger temperature changes also predicted higher confidence (β = 2.07, SE =  ±0.49, *P* = 0.00002). The direction of the temperature change was not a significant predictor of confidence (β = 0.22, SE =  ±0.13, *P* = 0.084).

## DISCUSSION

In this set of experiments, we investigated whether a temporal filtering mechanism enhances perception of simple and isolated decreases in noxious heat intensity, relative to isolated increases of a comparable size. We measured perception using a 2IFC task, or pain ratings of stimuli with unpredictable intensity changes. This allowed us to test for a sensory enhancement mechanism while controlling for any effects of expectation or response biases. Contrary to our prediction, in *experiment 1*, we found larger detection thresholds for decreases from 45°C than for increases from 45°C, and we replicated this finding in a different set of participants in *experiment 2*. Therefore, decreases in noxious heat intensity were more difficult to perceive than increases from the same initial temperature. Our results did not depend on whether the noxious stimulus was delivered using radiant or contact heat. This indicates that detection thresholds were not affected by differences in the biophysical mechanisms of radiant and contact heat stimulation (Iannetti et al. 2006) or by the unavoidable coactivation of mechanoreceptors with contact heat stimulation. Moreover, they were not affected by the rate of temperature change (2°C/s in *experiment 1* and 4°C/s in *experiments 2* and *3*).

We also found larger thresholds for discriminating the size of two decreases in noxious heat, compared with two increases in noxious heat. However, this difference in discrimination thresholds should be interpreted with caution. We had to exclude five participants from the discrimination threshold analysis because determining their increase discrimination thresholds would have required increasing the stimulus beyond the maximum safe limit of 50°C. Presumably, this meant that we excluded participants with relatively high increase discrimination thresholds, and this may have biased our result. Note, however, that the detection threshold result was unaffected by this issue.

In *experiments 1* and *2*, both increases and decreases in stimulus temperature always began at 45°C, so the temperatures used to find perceptual thresholds for decreases were always lower than those used to find perceptual thresholds for increases. In *experiment 3*, we repeated the detection threshold procedures using decreases that started from a higher initial temperature (47°C) than the increases did (45°C) so that the temperature ranges used in the two tasks overlapped. We found that decrease detection thresholds from 47°C were numerically larger than, but not significantly different from, increase detection thresholds from 45°C. This suggests that, within a common range of noxious temperatures, increases and decreases in noxious heat are perceived equally well. Furthermore, in experiment 2, we found that pain ratings of a 46°C stimulus preceded by a decrease from 47°C were no different than pain ratings of a stimulus that remained constant at 46°C for the same amount of time. Thus the prior decrease in stimulus temperature did not affect perceived pain intensity (nor did a prior increase from 46°C affect the perceived intensity of a 47°C stimulus).

**Fig. 5.**
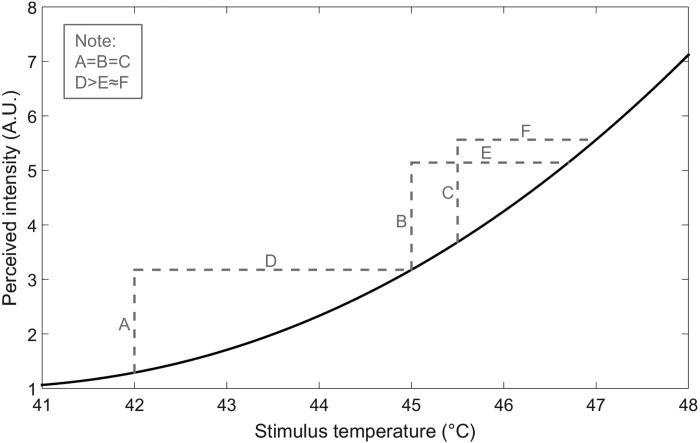
Hypothetical psychophysical stimulus-response function for heat stimulation going into the noxious range. A positively accelerating stimulus-response function could account for the finding (*experiments 1* and *2*) of larger perceptual thresholds for decreases from 45°C than for increases from 45°C (*A* = *B*; *D* > *E*), as well as the finding (*experiment 3*) of similar thresholds for decreases from 47°C and increases from 45°C (*B* = *C*; *E* ≈ *F*). A.U., arbitrary units.

Our findings are consistent with studies that have found positively accelerating psychophysical (Baliki et al. 2009; Coghill et al. 1993; Defrin and Urca 1996; Mørch et al. 2015; Nielsen et al. 2005; Price et al. 1983, 1994; Svensson et al. 1997; Yelle et al. 2008) and neural (Kenshalo et al. 1979; Price et al. 1978) stimulus-response functions for heat stimulation in the noxious range (>42°C) (although it should be noted that most studies of radiant heat stimulation tend to show a near-linear stimulus-response function; Adair et al. 1968; Hardy et al. 1952; Iannetti et al. 2008; Price and Browe 1973; Svensson et al. 1997). In addition, psychophysical studies of change detection in contact heat intensity found that both monkeys and humans could detect smaller temperature increments as the stimulus baseline increased from an innocuous level of 36–39°C to noxious levels of 46–47°C (Bushnell et al. 1983; Handwerker et al. 1982; Robinson et al. 1983). Figure 5 shows an example of how a positively accelerating stimulus-response function could have yielded our perceptual threshold results. In this function, from a starting temperature of 45°C, a much larger change in stimulus temperature would be required to reduce perceived intensity than to increase perceived intensity by an equal amount. On the other hand, increases in temperature from 45°C and decreases in temperature from 47°C would be perceived similarly, because they cross overlapping points on the stimulus-response function. However, our results should not be overinterpreted in this regard, because we did not directly measure psychophysical stimulus-response functions for our contact or radiant heat stimuli.

Our results provide clear evidence that there is no temporal contrast enhancement of simple and isolated decreases in noxious heat intensity, relative to increases of comparable sizes. We used a 2IFC design to specifically examine sensory processing of changes in noxious heat intensity while minimizing any effects of response biases or expectations on perceptual reports. Each noxious heat stimulus followed one of three stimulation profiles: *1*) a decrease from an initial level of noxious heat to a lower level, *2*) an increase from an initial level of noxious heat to a higher level, or *3*) a constant level of noxious heat with no change. Therefore, increases and decreases in noxious heat were always presented separately. This is different from the classic OA stimulation paradigm (Derbyshire and Osborn 2008, 2009; Grill and Coghill 2002; Honigman et al. 2013; Martucci et al. 2012a, 2012b; Nahman-Averbuch et al. 2014; Naugle et al. 2013; Niesters et al. 2011a, 2011b; Nilsson et al. 2014; Oudejans et al. 2015; Yelle et al. 2008, 2009), in which a slight decrease in noxious heat intensity is felt as disproportionately large when it is preceded by a slight increase in stimulus intensity from an initial noxious level. It has been proposed that OA results from a temporal filtering mechanism that enhances detection of noxious stimulus offsets (Grill and Coghill 2002; Yelle et al. 2008, 2009). Expanding on that proposal, some have further claimed that a temporal contrast mechanism might also enhance perception of simple and isolated decreases in noxious heat intensity that are not preceded by prolonged noxious stimulation or by increases from an initial level of noxious heat (Mørch et al. 2015; Yelle et al. 2008). Contrary to that particular claim, we found no evidence for enhanced perception of isolated decreases in noxious heat, relative to increases of the same size. Our result replicated across three experiments in separate groups of participants and did not depend on the kind of stimulus (contact or radiant heat), the rate of temperature change (2°C/s or 4°C/s), or the type of measurement (2IFC or pain ratings).

We did not directly compare our simple stimuli, consisting of individual increases or decreases in noxious heat, with the standard increase-decrease stimulation profile used to elicit OA. However, based on our findings and the differences between our stimuli and the standard OA protocol, we speculate that the initial increase in noxious heat may be key to the enhanced perception of the subsequent decrease. Alternatively, it may not be the increase per se, but the duration of noxious heat stimulation before the decrease that is important. The classic OA stimulation profile delivers at least 10 s of noxious heat stimulation before the temperature decrement, and a recent study found that a full 30 s of prior stimulation (15 s at the initial noxious level and 15 s at the higher level) was optimal for eliciting OA when the stimulus returned to its initial noxious temperature (Petre et al. 2017). This is consistent with other studies showing that the perceived intensity of a tonic noxious heat stimulus peaks around 10–15 s after stimulus onset before plateauing or reducing (Hardy at al. 1968; Koyama et al. 2004; Tran et al. 2010), and this plateau may involve thalamocortical modulation (Tran et al. 2010). Our stimuli, on the other hand, only delivered 1–5 s of noxious stimulation before the temperature change. We cannot rule out the possibility that longer durations of noxious heat stimulation might produce changes in central nociceptive processing that alter the temporal filtering of stimulus decreases, but we do show that such decreases, by themselves, are not perceptually enhanced.

In addition to measuring perception of changes in noxious heat intensity, we asked participants to judge how confident they were about each of their answers in the 2IFC tasks. We were interested in whether participants would report more (or less) confidence in their judgments about decreases in noxious heat, compared with their judgments about increases. People tend to be more confident in easy decisions than difficult ones (e.g., Baranski and Petrusic 1994; Gigerenzer et al. 1991; Griffin and Tversky 1992), and our perceptual threshold results showed that judgments about decreases were actually more difficult than judgments about increases. Therefore, a simple comparison between confidence judgments in increase and decrease threshold blocks would be confounded by task difficulty. To determine whether confidence might differ for judgments about increases and decreases in noxious heat, beyond any differences driven by task difficulty, we ran mixed models of confidence judgments. In *experiment 1*, participants were less confident in their judgments about decreases compared with increases, even after both accuracy and task difficulty were accounted for. In *experiments 2* and *3*, however, confidence was predicted by accuracy, but not by the direction of the temperature change. Although our confidence results are mixed, they suggest that participants are less confident in their judgments about decreases in noxious heat than in their judgments about increases. Importantly, this effect may not be fully accounted for by differences in the difficulty of these judgments.

Altogether, our findings demonstrate that people are better at detecting changes in noxious heat intensity within higher temperature ranges compared with lower ones. Within a common range of noxious temperatures, we found no advantage for detecting isolated decreases in stimulus intensity relative to isolated intensity increases. Moreover, pain ratings of a level of noxious heat at a particular moment did not depend on whether it was preceded by an unpredictable decrease from a higher temperature or by constant stimulation at the same temperature. These observations demonstrate that simple decreases in noxious heat stimulation are not subject to temporal contrast enhancement. Future studies may directly compare individual increases or decreases in noxious heat with the typical OA increase-decrease sequence, and with prolonged prior noxious stimulation without an increase from an initial noxious level, to determine the key stimulation parameters for eliciting perceptual enhancement of noxious stimulus offsets.

## GRANTS

This work was supported by Medical Research Council (UK) Project MR/M013901/1 (to P. Haggard and G. D. Iannetti). P. Haggard and G. D. Iannetti hold a residency at the Paris Institute of Advanced Studies. P. Haggard was additionally supported by European Research Council (ERC) Advanced Grant HUMVOL, and G. D. Iannetti by ERC Consolidator Grant PAINSTRAT and the Wellcome Trust Strategic Award COLL JLARAXR.

## DISCLOSURES

No conflicts of interest, financial or otherwise, are declared by the authors.

## AUTHOR CONTRIBUTIONS

B.B., G.D.I., and P.H. conceived and designed research; B.B. and S.G. performed experiments; B.B. and S.G. analyzed data; B.B., S.G., G.D.I., and P.H. interpreted results of experiments; B.B. and S.G. prepared figures; B.B. and S.G. drafted manuscript; B.B., G.D.I., and P.H. edited and revised manuscript; B.B., S.G., G.D.I., and P.H. approved final version of manuscript.
